# Family caregivers’ preferences for support when caring for a family member with cancer in late palliative phase who wish to die at home – a grounded theory study

**DOI:** 10.1186/s12904-024-01350-5

**Published:** 2024-01-11

**Authors:** Toril Merete Nysaeter, Cecilia Olsson, Tuva Sandsdalen, Reidun Hov, Maria Larsson

**Affiliations:** 1 Department of Health and Nursing Sciences, Inland Norway University of Applied Sciences, Elverum, 2400 Norway; 2 Department of Health Sciences, Karlstad University SE, Karlstad, Sweden; 3 Department of Bachelor Education in Nursing, Lovisenberg Diaconal University College, Oslo, Norway; 4 Centre for Development of Institutional and Home Care Services (USHT), Inland (Hedmark), Hamar, Norway

**Keywords:** Family caregiver preferences, Cancer, Palliative care, Home death, Grounded theory

## Abstract

**Background:**

Family caregivers are essential in end-of-life care for cancer patients who wish to die at home. The knowledge is still limited regarding family caregivers needs and preferences for support and whether the preferences change during the patient’s illness trajectory. Therefore, the aim was to explore family caregivers’ preferences for support from home care services over time when caring for a family member with cancer at the end of life who wished to die at home.

**Methods:**

A qualitative method was applied according to Grounded Theory. Data was collected longitudinally over the illness trajectory by means of repeated individual interviews (*n* = 22) with adult family caregivers (*n* = 11). Sampling, data collection and data analysis were undertaken simultaneously in line with the constant comparative method.

**Results:**

The findings are captured in the core category “hold out in duty and love”. The categories “having control and readiness for action” and “being involved in care” describe the family caregivers’ preferences for being prepared and able to handle procedures, medical treatment and care, and to be involved by the healthcare personnel in the patient’s care and decision making. The categories “being seen and confirmed” and “having a respite” describe family caregivers’ preferences for support according to their own needs to be able to persevere in the situation.

**Conclusion:**

Despite deterioration in the patient’s illness and the increasing responsibility family caregiver struggle to hold out and focus on being in the present. Over time together with deterioration in the patient’s illness and changes in the situation, they expressed a need for more intense and extensive support from the home care services. To meet the family caregivers’ preferences for support a systematic implementation of a person-centred care model and multicomponent psycho- educational interventions performed by nurses can be proposed. Moreover, we suggest developing a tool based on the conceptual model generated in this study to identify and map family caregivers’ needs and preferences for support. Such a tool can facilitate communication and ensure person-centred interventions.

**Supplementary Information:**

The online version contains supplementary material available at 10.1186/s12904-024-01350-5.

## Background

End-of-life care at home for patients with cancer is becoming more common due to an increasing number of persons who wish to die at home [1, 2], as home death is regarded by most as a more comfortable and dignified experience than death in an institution [3–6]. Family caregivers are essential to enable end-of-life care at home, a fact emphasised by research showing that home death is unlikely to be achieved without family caregivers [1, 7, 8]. However, caring for a family member at home is challenging and demanding, as persons near the end-of-life often have complex symptoms and needs that worsen as their illness progresses and death approaches [9]. According to the World Health Organization, palliative care is an approach aimed at improving the quality-of-life of both patients and their families facing problems associated with life-threatening illness [8]. Hence, consideration of family caregivers’ need for support is a crucial aspect of the quality of end-of-life care at home [10].

In the context of palliative care, a family caregiver is defined as someone close and significant to the patient, i.e. a partner, family member or friend who plays an important role in providing care and various forms of assistance to the patient [11, 12]. Family caregivers are considered corner-stones of palliative care by undertaking practical tasks, providing emotional support, and relieving pain and other symptoms, as well as communicating with health services, all with the purpose of improving the quality of life of someone close to them [13, 14]. Studies have shown that family caregivers experience a general duty of care [15], but that they are unprepared for the caregiving role [16, 17]. The process of long term caregiving to a close relative suffering from cancer is both physically and emotionally challenging [18], and family caregivers report various problems such as sleep disturbance, anxiety, depression, limitations to social life and financial challenges [19–22]. Studies show that family caregivers’ support needs vary during the illness trajectory [23] and the perceived caregiver burden has been shown to increase in line with the patient’s deteriorating health status, increasing demands for symptom management and need for end-of-life and terminal care decisions [21, 24]. Furthermore, family caregivers have a close relationship with the patient, and are involved in the process of dying and the prospect of death. Consequently, the physical and emotional burden of caregiving is often extensive [21].

There are several studies describing and evaluating different types of support to family caregivers who are caring for an end-of-life patient at home [25–31] and instruments have been developed to identify and map family caregivers’ various needs for support [32–34]. However, the knowledge base is limited regarding family caregivers’ preferences for support, and especially whether and how the preferences change during the patient’s illness trajectory. Hence, in order to support family caregivers in managing the situation during the entire care trajectory and to enable death at home in accordance with the patient’s wishes, additional knowledge is needed regarding their preferences for support. This kind of knowledge is essential in order to design person-centred supportive interventions that are responsive to changes in family caregivers’ preferences for support [35, 36]. Therefore, in this study we aimed to explore family caregivers’ preferences for support from home care services over time when caring for a family member with cancer at the end of life who wished to die at home.

## Method

### Design

An exploratory design using grounded theory was used [37]. Data was collected longitudinally in repeated interviews one to four times during the illness trajectory. The present study is reported in accordance with Consolidated criteria for reporting qualitative research (COREQ) recommendations [38].

### Setting, participants and recruitment

We performed a study exploring dyads of patients’ and family care givers’ preferences for care and support over time to enable home death. The study was performed in six different municipalities, both urban and rural, in two different communities in the southeast of Norway. The study setting and recruitment have previously been described together with data from the patients’ perspective, by Nysaeter et al. [39]. All six municipalities have a cancer nurse employed as a cancer care coordinator. Cancer care coordinators play an important role in the municipalities in Norway, as they coordinate care and are responsible for providing continuity and support to patients and their family caregivers throughout the trajectory of care [40]. In the present study, data from interviews with family caregivers in nine dyads is used, together with data from two additional bereaved family caregivers. Hereafter, the term “patient” is used about the family member with cancer who is receiving end-of-life care at home.

Family caregivers were invited to take part in the study when they met the following inclusion criteria: being or having been a family caregiver to a patient with cancer at the end of life with expected death within 6–12 weeks; being informed and aware of the patients’ state of illness and prognosis; being adult (at least 18 years of age); having no cognitive impairment; understanding and speaking Norwegian; and cohabitating with the patient or living in their own home in a geographical proximity to the patient. Family caregivers to patients living in nursing homes were excluded.

The cancer care coordinators informed and recruited dyads of patients and family caregivers, and the two bereaved family caregivers. Eligible patients gave consent for the family caregivers to participate. When a family caregiver agreed to participate, the cancer care coordinator informed the first author (TN), who contacted the family caregiver, clarified any questions about the study and made an appointment for the time and place of the first interview.

All family caregivers who were invited gave their consent to participate (*n* = 11) (Table 1). The family caregivers’ characteristics are described in Table 1. At the time of their recruitment all family caregivers had been informed about the cancer care coordinator’s role and had been given the opportunity to receive support from the cancer care coordinator. Three of the family caregivers received support from a cancer nurse in the palliative care team at the hospital.

**Table 1:** Characteristics of participants (*n* = 11)

Characteristics	Number
**Sex**	
Women	10
Men	1
**Age (years)**	
Median	64.3
Range	42–85
**Marital status**	
Married / cohabitant	8
Living alone	3
**Education level**	
Elementary school	5
Secondary school	3
University college / university	3
**Living conditions**	
Detached house	7
Semi detached / multi-storey building	4
**Ethnicity**	
Ethnic Norwegian	11

### Data collection

Data collection was performed as individual interviews between April 2018 and June 2019, together with two additional interviews conducted in December 2021. The family caregivers had the opportunity to choose the arena for the interviews. Most of the interviews were conducted in the family caregiver’s home, except for three. Out of these, two were held in a private room at the researcher’s workplace and one at hospital.

An interview guide was developed by the authors drawing on existing evidence and clinical knowledge of the field, with topics concerning the family caregivers’ support preferences, what kind of support they received, how they experienced the support, whether they got the support they preferred and how it was consistent with their wishes and needs for future support [37].

The first author (TN) conducted, and audio recorded all interviews. Each interview started as an open dialogue in which the family caregivers was encouraged to speak freely about their experiences of being a caregiver and preferences for support regarding home care services. The interview guide was used as a reminder during the interview, to ensure that the topics listed above were covered. Open questions such as who, which, what, etc. were asked in order to extend or narrow the field of interest. In the follow up interviews the interview guide was changed and modified between each interview focusing on broadening and clarifying the emerging categories, their properties and dimensions [37]. In addition, the follow up interviews also contained questions exploring potential changes in family caregivers’ preferences for support since the last interview.

From the beginning, the sampling was purposeful in accordance with the inclusion criteria for both patients [39] and family care givers in the dyads. As the inclusion process proceeded, theoretical sampling of dyads was primarily used to saturate the emerging categories in the patient study. The data from the interviews with the family caregivers in these dyads was predominantly characterised by negative experiences. Therefore, theoretical sampling of family caregivers continued, with the intention of challenging the preliminary results, and two additional bereaved family caregivers, who had expressed positive experiences of end-of-life care at home, were interviewed once.

The family caregivers (*n* = 11) were interviewed from one to four times (Table 2). In total, 22 interviews were conducted. The follow-up interviews were held when there was a deterioration in the patient’s illness status, such as the onset of distressing symptoms, pain that needed professional management, infections, or self-reported experience from the patient that death was approaching. When such a change occurred the family caregiver contacted the first author (TN) and made an appointment for a follow-up interview. The only male participant was interviewed once. Five female participants in the longitudinal study also attended an interview after the patient’s death. The time between the initial interview and the follow-up interviews varied between one and three weeks. A final follow-up interview was held two weeks up to two months after the patient’s death with five of the family caregivers in the dyads. The two additional bereaved family caregivers were interviewed three and nine months, respectively, after the patient’s death.

The interviews (*n* = 22) lasted from 25 min up to 75 min (median 30.5 min). In total 21 h and 14 min of interviews were analysed. After each interview, field notes were written to capture the immediate thoughts and impressions from the interview.

**Table 2:** Overview of the number of interviews per participant over time

Participants	Interview 1	Interview 2	Interview 3	Interview 4*
Family caregiver 1	x	x	x	x
Family caregiver 2	x			
Family caregiver 3	x			x
Family caregiver 4	x	x		x
Family caregiver 5	x			
Family caregiver 6	x	x		x
Family caregiver 7	x			
Family caregiver 8	x	x	x	x
Family caregiver 9	x			
Family caregiver 10				x
Family caregiver 11				x

*Interviews conducted after the patient’s death

### Data analysis

Data collection and data analysis were performed as a cyclical process in line with the constant comparative method in grounded theory [37, 41]. The first author (TN) transcribed the interviews verbatim, as close to the interview as possible. Field notes were included in the memos. The open coding process started after reading through the interview and memos. The open coding process were performed by breaking the text, interviews and memos apart and delineating concepts into preliminary categories. To ensure closeness to the data, the coding process were performed by hand by the first author (TN) and discussed in the research group.

In the coding process memos were included (TN). Questions such as “what, where, who, when and what consequences” were asked of the text and contributed to clarifying the categories. The axial coding procedure, which contains a seek for crosscutting and relations between categories, led to further clarification of the categories (TN, CO, TS, RH, ML). In the selective coding process, each category was saturated with information from new interviews or already analysed interviews.

According to the method, memos were written by the first author (TN) during the analysis process and shared with the research group to ensure that thoughts and reflections were taken care of. Finally, a core category was identified, and a conceptual model was established.

## Results

**Fig. 1:**
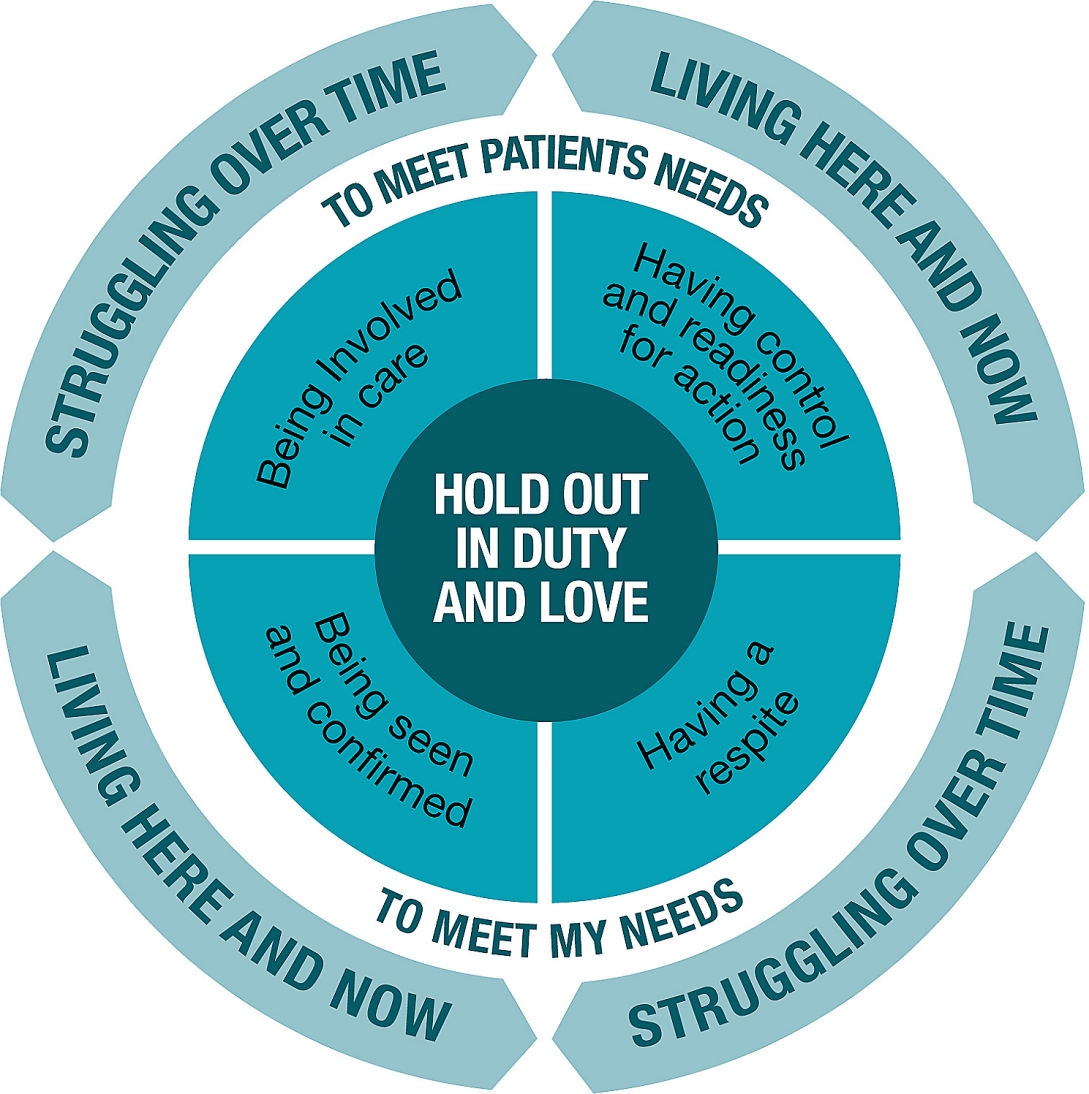
Conceptual model of the preferences for support from home care services over time among family caregivers caring for a family member with cancer at the end of life who wished to die at home

The findings are captured in the core category “hold out in duty and love”. The core category shows that despite deterioration in the patient’s illness and the increasing responsibility, they carry on in their role as family caregiver, and they struggle to persevere and focus on being present in the moment. The preferences for support from home care services were divided between preferences related to care and support for the patient, and preferences for support for themselves, as illustrated, see Fig. 1. The family caregivers stressed that in everyday life, the patient’s wellbeing comes first, and their own needs are secondary. Most important was that the patient got the best care and support from home care services, which was also considered to be support for themselves. Secondly, the family caregivers need support from the home care services to be able to persevere over time. The categories “having control and readiness for action” and “being involved in care” describe the family caregivers’ preferences for being prepared and able to handle procedures, medical treatment and care, and to be involved by the healthcare personnel in the patient’s care and decision making. The categories “being seen and confirmed” and “having a respite” describe family caregivers’ preferences for support in accordance with their own needs, to be able to persevere in the situation. Over time, along with deterioration in the patient’s illness and changes in the situation, they expressed a need for more intense and extensive support within each category.

## Hold out in duty and love

The family caregivers describe that they experience being close to a patient with cancer as like living in a bubble captured by the cancer illness. Being the person closest to the patient, they experience having a great responsibility for both the patient’s wellbeing and for the care of the patient. Loyalty and commitment permeated the interviews. They described a strong wish to be there for the patient due to an emotional bounding, or a promise they had once made, to fulfil the patient’s expressed wish to be cared for and to die at home. Some of the family caregivers expressed how when they had given their promise, they were not aware of how demanding the situation would be. Throughout the patients’ illness trajectories, they experienced contradictory and mixed feelings. Feelings of exhaustion, anger and fear for the future challenged their ability to persevere. On the other hand, feelings of joy and togetherness contributed to their persistence.

They describe how, that to be able to persevere, they focus on living here and now and taking one day at a time, aiming to achieve good moments together with the patient. Good moments are described as moments together, experiencing a feeling of normality. At the same time, they are aware that the illness is progressing, and the loss of the patient is becoming imminent. The preference of having someone close to talk to and the importance of a continuous relationship were described as necessary in order to persevere.

### Having control and readiness for action

The family caregivers described preferences for having control of the situation and a readiness for action when needed. One important preference was predictability regarding the delivery of care from the home care services and the cancer coordinator, as this enabled them to plan their day. To be able to have control and to be prepared, they preferred honest information about the prognosis of the illness and what changes they could expect due to a deteriorated condition. Being informed also made them able to judge and manage acute situations that could arise including knowing whom to contact 24/7 depending on the severity of the situation.

*“Just before Easter he got ill and I thought that he had a urinary tract infection. Then I called the cancer care coordinator and told her about the symptoms. All right, she says, we’ll send him to the hospital, for treatment. I called the ambulance, and it arrived at 11 o`clock.”*

*Interview 1, family caregiver 1, aged 75.*

This becomes more important as the illness progresses and death becomes imminent. To be ready for action, they also wanted to have knowledge about the patient’s medication, treatment and care. In addition, they expressed a preference for practical training in procedures such as pain management and wound care, but also fundamental care such as personal hygiene and nutrition.

*“I got trained in using that machine and so did he. If anything appeared we were able to manage ourselves. It was only two or three times that we had to do it ourselves”.*

*Interview 1, family caregiver aged 56.*

### Being involved in care

The family caregivers underlined that they wanted to be involved in the care of the patient and accepted as part of the home care team. This preference was especially pronounced among those who were living together with the patient.

*“We were a team working well together and I felt accepted as a part of the team. I did what I could do…. It became natural and comfortable and maybe that’s why I felt it (the situation) not so burdensome”.*

*Interview 1, family caregiver 11, aged 66.*

Early in the illness trajectory, most family caregivers preferred to care for the patient themselves. When the patient’s healthcare needs became more severe and the home care services had to be involved, they preferred shared care responsibility. They wanted to contribute their experience and knowledge about the patient in their cooperation with the healthcare personnel.

*«In an ideal world I would wish that I had been included more, earlier.”*

*Interview 2, family caregiver 4, aged 48.*

The understanding of the patient’s situation, the severity of the illness and that death was approaching was facilitated by being involved in the direct care of the patient, as well as decisions regarding care and treatment. This was evident in the interviews after the patient’s death, where the family caregivers stated that they wished that the healthcare personnel had involved them in the patient’s care to a greater extent.

### Being seen and confirmed

Family caregivers expressed a preference to be seen as a person with their own need for support, both to an increasing extent during the course of care and for a period after the patient’s death. In addition, they described a preference to be acknowledged for their efforts and the care they provided, but they often experienced being taken for granted.

*«Just to be seen and experience that someone was interested in ME, and my story and my role in it, and how tough it actually is for me being his family caregiver. That was incredibly good.”*

*Interview 2, family caregiver 4, aged 48.*

Family caregivers expressed a preference to share feelings they were ashamed of, such as anger and irritation that a patient’s illness persisted 24/7. In meetings with healthcare personnel, they preferred those who showed empathy, took the time to listen, and took the initiative for conversations.

*“I think it is frustrating that illness has taken over everything, in a way taking over life. And I don’t want it to be like that.”*

*Interview 1, family caregiver 5, aged 44.*

They also describe a need to talk to someone about feelings of fear of the future and the imminent loss of the patient. Often family caregivers had these talks with someone close to them. However, after the patient’s death they expressed a preference to talk to competent and empathetic healthcare personnel. Family caregivers preferred to talk to cancer care coordinators, with whom they had an established relationship. In addition, they were considered especially competent regarding end-of-life care and bereavement.

### Having a respite

To be able to hold out and manage to live in “the cancer bubble”, the family caregivers described a preference for respite from the situation. Having a respite meant having time alone, for example going out for a walk or going to the grocery store and knowing that the patient was safe at home. This was achieved by either an alarm to the home care services or planned visits with a known, trustworthy and competent person. Family caregivers needed the health care personnel to encourage them to leave the home, because they could feel guilty for taking time on their own and not being available for the patient 24 h a day.

*“It’s a situation where you gradually become exhausted if you don’t take care of yourself… It’s important to get out of the house.”*

*Interview 1, family caregiver 2, aged 85.*

For those not living with the patient, constant worry was common. For them, respite meant to be assured that the patient was safe and got the care and support they needed from home care services. Another aspect of respite care was being able to do things that enabled them to forget their caregiving responsibilities for a moment providing a sense of normality. This might be leaving home and to spend some hours at work. However, it could also mean having moments together with the patient, without thinking of the illness. This might entail doing things they used to do before the illness, such as enjoying time in the garden, having dinner or watching TV together.

*“I want us to achieve more good moments together, go out for a walk with the neighbour’s dog. Watch TV, such a simple thing as watching a programme together. Such things, it is happiness.”*

*Interview 1, family caregiver 6, aged 55.*

## Discussion

This study explored family caregivers’ preferences for support from home care services over time when caring for a family member with cancer in the late palliative phase who wished to die at home. End-of-life care was a challenge for the family caregivers, and they struggled to fulfil the patient’s wish to die at home out of loyalty and commitment. The preferences for support to be able to persevere were found to consist of two interrelated parts - preferences related to the patient’s need for care and support, and preferences for support for the family caregivers themselves. However, the family caregivers stressed that the patients’ needs and wellbeing were more important than their own. Consequently, when the patients’ needs for care and support were met, they experienced this as support for themselves, too. A significant finding of our study was that the family caregiver’s preferences for support did not change over time regarding its constituent parts, but to the extent to which support was provided. Accordingly, to persevere over time, concurrently with a deterioration in the patient’s illness, the family caregivers expressed a need for more intensive and comprehensive support for what they already received from home care services. It is well-known that feelings of carrying a burden and unmet needs among family care givers are associated with poorer health and quality of life [42, 43]. Accordingly, the findings of our study underline the importance of continuous assessment of both the patients’ and family caregivers’ needs, and the provision of adequate support throughout the care trajectory, in order to enable end-of-life care and death at home.

The family carers in our study expressed strong preferences for having control, a readiness for action and being prepared for the role as family caregiver, including what to expect as the patient’s illness deteriorated. Previous research has shown that caregiving preparations among family caregivers caring for a family member suffering from a progressive life-threatening illness are an ongoing process throughout the entire illness trajectory and intimately linked to preparedness for death and bereavement [44]. Family caregivers who feel prepared experience caregiving as less burdensome [45, 46] and tend to experience more hope and caregiving rewards. Moreover, preparedness has been shown to reduce negative outcomes such as poor health and quality of life [47, 48]. Preparedness requires involvement and it is well-known that family caregivers want to be involved in the care and that their contribution to palliative home care is substantial in terms of undertaking practical tasks, providing emotional and social support and relieving symptoms [13, 14]. In our study we also found that the family caregivers had a strong preference and wished to be involved in the care of the patient, both in terms of care decisions and practical care. To achieve this, the family caregiver must be acknowledged as a partner in care and training in the necessary medical procedures provided, such as for example administrating pain pumps.

Involvement in care was also stressed by the participants in our study as being of great importance for the family caregivers to be able to persevere over time. Being involved in care meant being seen and confirmed as a member of the care team around the patient, and also as someone with their own support needs, which is in line with the palliative care philosophy, i.e. seeing the family as a unit of care [8, 49]. Hence, the family caregivers expressed a wish for collaboration, to be provided with sufficient and clear information, and for healthcare personnel to be continuously available and accessible [14]. Moreover, our study highlights timing of involvement of family carers in the care process as crucial, as some participants stressed that they wished that they had been involved earlier in the patient’s care. Family caregivers also expressed that being involved as a member of the care team was experienced as sharing the care responsibility with someone with professional expertise. These finding underline the importance of involving family caregivers systematically from the beginning of the caregiving period aiming to decrease the caregiver burden and enable home death. Earlier studies have shown that the caregiver burden increases in line with the length of the caregiving period, and the patient’s declining health status, with increasing demands for symptom management and needs for decisions regarding terminal care [21, 43]. It is therefore of the utmost importance that not too much responsibility is placed on the family caregiver as the risk of exhaustion is evident. Hence, supportive interventions adapted to the evolving situation and individual characteristics are necessary, as recently stressed in a systematic review [50]. The family caregivers in the present study emphasised that they felt responsible for fulfilling the patient’s wish to be cared for and die at home. The fact that the vast majority of the family caregivers were women may have had an impact on how they took on the role as a caregiver, the burden they experienced and their preferences for preparedness. Previous research highlights that female caregivers exhibit a greater sense of responsibility towards the patient and generally experience lower levels of caregiver burden [48, 51, 52]. However, the family caregivers in the present study described a preference for having a respite from caregiving. Thus, in line with other research [53] the findings of the present study stress the importance of recognising the family caregiver’s needs for relief, adjusted to the caregivers’ preferences.

This study adds to the knowledge base regarding palliative home care and highlights the need for interventions targeting family carers’ needs, based on their preferences for support to be able to fulfil the patient’s wish to die at home. Given the growing number of people with progressing life-threatening illnesses who will be in need of palliative care in the coming years, support for family caregivers should be a priority area of care. Positively, studies evaluating supportive interventions for family caregivers in palliative home care are becoming more common, showing great potential for enhancing care giving expertise and preparedness for caregiving, while reducing the caregiver burden [50]. However, the studies evaluating the interventions, often categorised as psychosocial, educational or psycho-educational, largely lack scientific rigour, limiting the interpretation of their effectiveness [28]. Nevertheless, systematic reviews highlight that multicomponent interventions performed by nurses are efficient and have positive effects on family caregivers’ outcomes [50, 54]. In particular, nurses who adopt person- and family-centred care and frequently communicate with the family caregivers, are in a unique position to provide adjusted support. The evidence from these studies can be used to develop the cancer care coordinator role in Norway as these coordinators have the opportunity to follow the cancer patient and the family caregivers throughout the illness trajectory.

### Methodological considerations

As sincerely as possible, we have sought to follow Corbin and Strauss‘ criteria for evaluating the quality of research of a GT-study [37]. The list of criteria concerns the research process and the empirical grounding of the study and comprises clear descriptions of the sampling procedures, the constant comparison, concept generation, emerging categories, selection of the core category, concept generation and variation in theory. We have sought to give a clear description of the process in the description of the method. Throughout the whole research process there has been a close collaboration in the research group, which has contributed to ensure the reliability of the findings. During the data analysis we have discussed our own preconceptions and various preconceptions about the topic, and we have used critical thinking to ensure that the categories were grounded in data and not reflected our preconceptions. We have also used memos to maintain an audit trail of the research process [41].

There are some limitations to the study. One limitation to be considered is the sample size (*n* = 11) as it might be judged as small. At the beginning of the study, the sampling was purposeful, as the cancer care coordinators asked patients and thereafter family caregivers who fulfilled the inclusion criteria. The first interviews (*n* = 9) were pre-dominantly characterised by negative experiences. Therefore, we used theoretical sampling, and two additional bereaved family caregivers were recruited. The additional interviews validated and gave breadth to the result, and densified the categories, and the axial- and selective coding process could be carried out successfully, while the number of family caregivers finally included might be considered as appropriate.

The temporal aspect of this study might also be questioned as only five family caregivers were interviewed more than once. Three of the family caregivers included were interviewed once, as they either declined a follow up interview due to deterioration in the patient’s illness [2], or did not answer the request after the patient’s death [2]. However, in total 22 interviews were conducted, which created rich data material, and which could be considered appropriate and therefore mirroring family caregivers’ preferences for support from home care services.

The skewed gender distribution of the family caregivers, as ten women and one man, should be mentioned. However, in the recruitment process we recruited family caregivers who met the criteria of being a family caregiver to a patient who had a wish to die at home, and these patients were mostly men. This is in line with previous studies showing that more men than women die at home [55, 56].

As data from additional interviews did not contribute to new categories, this was interpreted as a sign of category saturation. The research group discussed this in depth and agreed that saturation had been reached. In addition, before and during the data analysis the research group discussed their own preconceptions about the subject in focus and used critical reflection throughout the research process in order to find the hidden meaning. The first author, TN, is a specialist nurse in cancer care (CN), educated and trained in Clinical Supervision and Communication. As the first author she were close to the family caregivers throughout the interviews and her role was examined thoroughly by the research group according to the preconditions.

## Conclusion

Family caregivers’ preferences for support from home care services were twofold. On the one hand, there were preferences related to end-of-life care and support for the patient, and on the other hand, preferences for support for themselves to persevere over time. The family caregivers stressed that in everyday life the patient’s well-being came first and their own needs second, and when the patient’s needs for care and support were met, they experienced this as support for themselves, too. Hence, the family caregivers main concern was that the patients’ needs for care and support were met. In our study, the family caregivers’ support preferences did not change over time regarding their constituent parts, but in terms of the extent to which support was provided. Over time, together with deterioration in the patient’s illness and changes in the situation, they expressed a need for more intense and comprehensive support. Therefore, we stress the need for continuous assessment of both the patients’ and family caregivers’ needs throughout the care trajectory and adjusted supportive interventions based on their preferences, in order to enable end-of-life care and death at home. Multicomponent interventions performed by nurses are suggested, thus enabling a person- and family-centred end-of-life home care.

We also suggest developing a tool to map family caregivers’ needs and preferences for support based on the conceptual model generated in the current study. Such a tool could facilitate communication between family caregivers and healthcare personnel and constitute a basis for timely person-centered interventions.

### Electronic supplementary material

Below is the link to the electronic supplementary material.

**Supplementary Material 1:** Interview guide

## Data Availability

The datasets generated or analysed during the current study are not publicly available due to the participants did not include sharing the dataset as well as the conditions set by NSD (Norwegian Centre for Research Data) but are available from the corresponding author on reasonable request.
