# Supplementary material for: Family caregivers’ preferences for support when caring for a family member with cancer in late palliative phase who wish to die at home – a grounded theory study
Source: BMC Palliat Care. 2024 Jan 11;23:15. doi: 10.1186/s12904-024-01350-5 (PMC10782637; doi:10.1186/s12904-024-01350-5)
Supplement: Supplementary file 1 — Supplementary Material 1: Interview guide [file 12904_2024_1350_MOESM1_ESM.docx]

**«Family caregivers’ preferences for support»**

**Interview guide**

1. Please, can you tell me about how is it to be a family caregiver?
2. Can you tell me about your preferences for support from the home care services?
3. What kind of support do you receive?
4. How do you experience the support?
5. Do you get the support you prefer?
6. Do you get the support you need?
7. Is the support consistent with your wish and need for support?
8. What are your thoughts about the future?

Using probing questions such as who, which, what, etc.
